# Gastrointestinal amyloidosis in a 50‐year‐old patient with miliary tuberculosis: A case report

**DOI:** 10.1002/ccr3.8978

**Published:** 2024-05-24

**Authors:** Sameer Abdul Rauf, Hussain Haider Shah, Rahul Khatri, Mansoor Ul Haq, Tirth Dave, Javaria Parwez Ali, Syed Khizar Ali

**Affiliations:** ^1^ Department of Internal Medicine Liaquat National Hospital and Medical College Karachi Pakistan; ^2^ Department of Internal Medicine Dow University of Health Sciences Karachi Pakistan; ^3^ Department of Gastroenterology Liaquat National Hospital and Medical College Karachi Pakistan; ^4^ Bukovinian State Medical University Chernivtsi Ukraine; ^5^ Department of Histopathology Liaquat National Hospital and Medical College Karachi Pakistan

**Keywords:** amyloidosis, gastrointestinal amyloidosis, miliary tuberculosis, tuberculosis

## Abstract

This case highlights the importance of considering tuberculosis as an underlying cause of gastrointestinal amyloidosis, even in patients previously treated for the infection. Clinicians should maintain a high index of suspicion for atypical presentations of amyloidosis, especially in individuals with chronic inflammation, enabling early diagnosis and tailored management for improved patient outcomes.

**Abstract:**

Gastrointestinal amyloidosis is a rare condition often associated with chronic inflammation. We present a unique case of a 50‐year‐old female with a history of miliary tuberculosis who developed gastrointestinal amyloidosis. The patient exhibited chronic loose stools, weight loss, abdominal pain, and urinary incontinence symptoms. Diagnostic workup revealed characteristic findings of amyloidosis on biopsy. Despite treatment for tuberculosis, her symptoms persisted, highlighting the challenging nature of managing this condition. This case underscores the importance of considering tuberculosis as a potential cause of secondary amyloidosis in patients with ongoing symptoms of inflammation and infection. Early recognition and tailored management are crucial in optimizing patient outcomes.

## 1 INTRODUCTION

Amyloidosis is a rare disease characterized by the extracellular deposition of abnormal proteins in various tissues and can be classified into six types: primary (systemic), secondary (systemic), hemodialysis‐related (systemic), hereditary (systemic), senile (systemic), and localized.^1^ Secondary amyloidosis involves the deposition of fibrils containing serum amyloid A (SAA) protein, a liver‐produced protein generated in response to persistent inflammation. These fibrils deposit within various organs, causing dysfunction and giving rise to clinical symptoms.^2^ The development of secondary amyloidosis is closely associated with chronic inflammatory or infectious diseases. Conditions such as rheumatoid arthritis, ankylosing spondylitis, inflammatory bowel disease (Crohn's disease and ulcerative colitis), chronic osteomyelitis, and tuberculosis have been recognized as common causes.^3^ These chronic diseases create a persistent inflammatory milieu that triggers the production of SAA protein, leading to amyloid deposition.^4^, ^5^ Secondary amyloidosis typically involves the kidneys, liver, spleen, and adrenal glands, but gastrointestinal involvement is rare.^6^ Here, we present a unique case of gastrointestinal amyloidosis associated with miliary tuberculosis.

## 2 CASE HISTORY/EXAMINATION

A 50‐year‐old female presented with a history of urinary incontinence for the past year, weight loss of 5 kg and loose stools persisting for the last 2 months, fever of 103–104 degrees Fahrenheit 2 weeks ago, and on‐and‐off abdominal pain. On general physical examination, the patient initially appeared lethargic and dehydrated on admission but has since shown improvement. Body temperature, blood pressure, respiratory rate, pulse, and SPO2 were afebrile, 120/75 mmHg, 20 bpm, 86 bpm, and 96%, respectively.

According to the patient's attendant, the patient was diagnosed with miliary tuberculosis (TB) based on a bone marrow biopsy in November 2022. She received anti‐tuberculosis treatment (ATT) for 6 months. However, approximately 50 days ago, she developed persistent loose stools (3–4 episodes per day) with a watery consistency. She stopped taking ATT and opted for symptomatic treatment for her loose stools. 2 weeks ago, she experienced a high‐grade fever (103–104 degrees Fahrenheit), relieved by medication. She also reported experiencing abdominal pain.

## 3 METHODS (DIFFERENTIAL DIAGNOSIS, INVESTIGATIONS, AND TREATMENT)

During her previous admission, multiple investigations were performed, including abdominal ultrasound (USG), which showed an enlarged liver, coarse and thickened, mild splenomegaly, fluid streak in the pelvis, distal stomach thickening, and bowel loop thickening. CT scan of the abdomen and pelvis showed minimal ascites, minimal splenic enlargement, abdominal lymphadenopathy with enlarged nodes in the mesenteric region, mesenteric thickening, and dilated bowels. Endoscopy revealed reflux esophagitis. Colonoscopy: This was unremarkable. Rectal biopsy showed mild‐to‐moderate inflammation with local cryptitis. Interferon‐gamma release assay (IGRA): Negative.

## 4 CONCLUSION AND RESULTS (OUTCOME AND FOLLOW‐UP)

**FIGURE 1:**
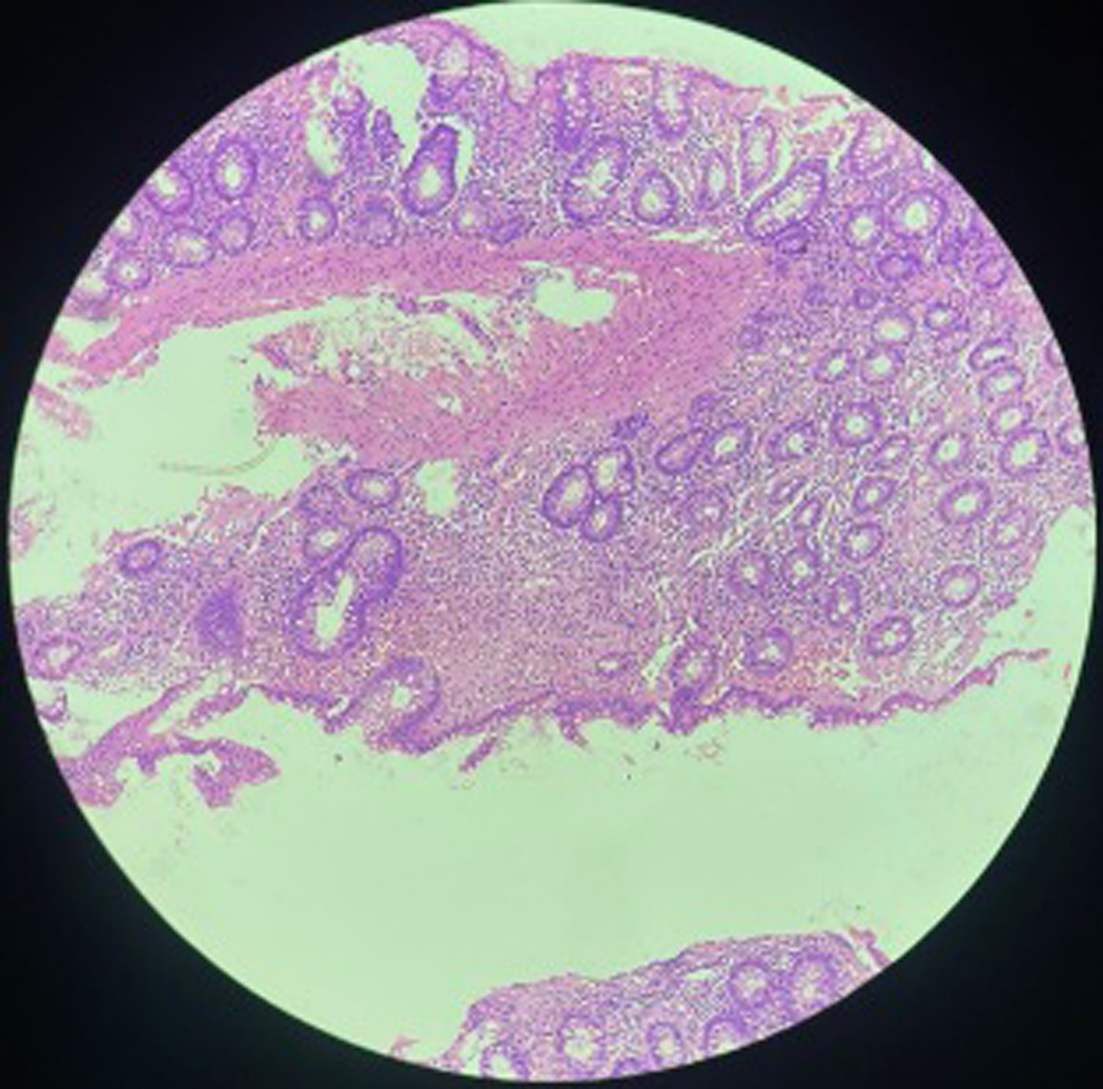
Fragments of small and large bowel mucosa. The small bowel mucosa exhibits partial villous blunting, with the lamina propria and submucosa displaying multiple foci of eosinophilic material.

**FIGURE 2:**
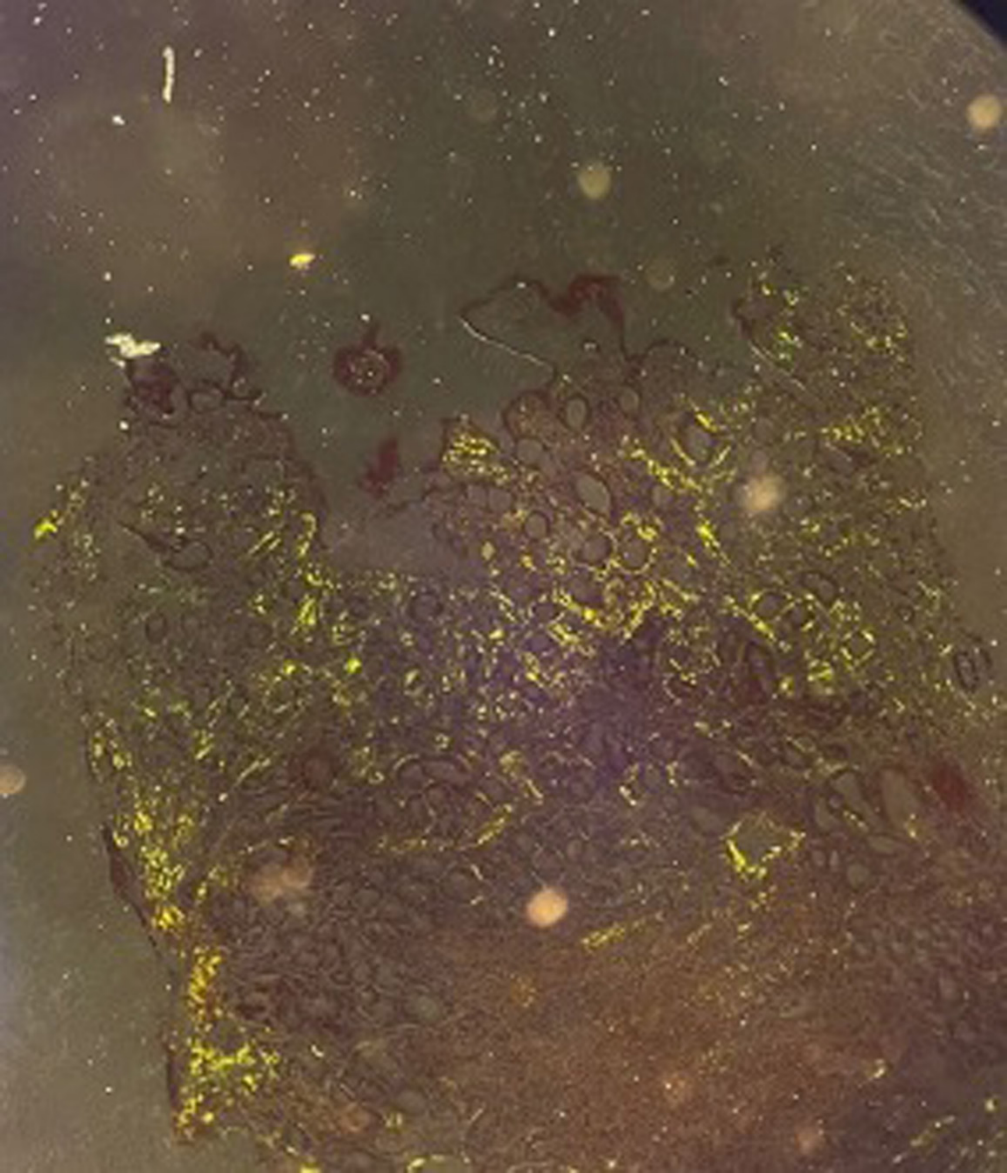
Fragments of small and large bowel mucosa stained with Congo red, revealing green birefringence under polarized light in these regions.

During her current hospital stay, the patient's electrolytes were found to be deranged (Table 1) and continuous replacements were administered. Nephrology was consulted for electrolyte management. The infectious disease department restarted ATT. Further investigations included stool studies and ascitic fluid studies. Stool culture and sensitivity (C/S) findings initially showed Vibrio, but later Gram‐negative rods were identified. Ascitic fluid studies were regular. Gastroenterology performed endoscopy and colonoscopy, and biopsy samples were sent for histopathology, which suggested acellular eosinophilic material with amyloidosis (Figures 1 and 2).

**TABLE 1:** Blood reports of the patient.

Item	Results	Normal range
Hemoglobin (g/L)	7.23 g/dL	12–16 g/dL
Hematocrit	7.17%	36%–48%
Platelets	100–150 × 10^9^/L	150–400 × 10^9^/L
Calcium (Ca)	9.57 mg/dL	8.7–10.2 mg/dL
Phosphate (PO_4_)	0.94 mg/dL	2.8–4.5 mg/dL
Magnesium (Mg)	2.01 mg/dL	1.7–2.2 mg/dL
Serum albumin	1.82 g/dL	3.4–5.4 g/dL
Serum globulin	4.35 g/dL	2.0–3.5 g/dL

Drug history includes loperamide twice daily, Saccharomyces boulardi twice daily and oral rehydration solution (ORS) as needed. The patient also received 3–4 pints of packed cell volume (PCV) transfusion 2–3 months ago. An additional 2 PCVs were transfused during this hospital stay. The patient continues to have persistent loose stools and a fever once every 24 h. The family has been counseled, and the patient is now being discharged.

## 5 DISCUSSION

Gastrointestinal amyloidosis has a very low incidence. The global incidence of amyloidosis is 5 per million person‐years.^7^ Congo red staining is a standard and practical test used to differentiate amyloid deposits from other protein deposits.^8^ Secondary amyloidosis is a type of amyloidosis that occurs due to chronic inflammation or infection.^9^ In this case, the patient's history of miliary tuberculosis and chronic loose stools suggest that secondary amyloidosis may have developed due to the chronic inflammation caused by tuberculosis.

Ninety percent of patients developed profound fatigue, weight loss, and edema. Edema may have multiple causes, including hypoalbuminemia, right‐heart failure, or simply the impairment of the blood vessels' ability to handle hydrostatic pressure challenges. Other symptoms depend on the involvement of the particular organs. In 15–25% of patients, liver involvement was found; in 15–20%, neuropathy was seen, and cardiac involvement was up to 50%.^10^ In this case, the patient's symptoms of loose stools, abdominal pain, weight loss, and urinary incontinence are consistent with the clinical presentation of gastrointestinal amyloidosis. The presence of electrolyte imbalances and fever also suggest ongoing inflammation and infection.

The diagnostic workup for gastrointestinal amyloidosis includes imaging studies, endoscopy, and biopsy. The gold standard test to establish a diagnosis of GI amyloidosis is tissue biopsy.^11^, ^12^ No specific treatment currently exists for managing gastrointestinal amyloidosis. Managing gastrointestinal amyloidosis depends solely on the underlying cause and the extent of organ involvement. Dietary modification, prokinetic agents, parenteral nutrition, anti‐diarrheal agents, antibiotic therapy, and management of GI bleeding were considered according to the presenting symptoms.^13^, ^14^, ^15^ The prognosis for gastrointestinal amyloidosis depends on the extent of organ involvement and the underlying cause.^16^

Overall, this case highlights the importance of considering tuberculosis as a potential cause of gastrointestinal amyloidosis, particularly in patients with a history of tuberculosis and ongoing symptoms of inflammation and infection. Further investigations and ongoing management are necessary to determine the extent of organ involvement and optimize the patient's prognosis.

This case underscores the complexity of gastrointestinal amyloidosis, especially in the context of underlying tuberculosis. The challenge lies in its varied presentation and the need for a high index of suspicion. Despite treatment for tuberculosis, the persistence of symptoms highlights the intricate nature of managing this condition. Clinicians must remain vigilant, considering unusual etiologies in chronic inflammatory states, ensuring timely diagnosis, and initiating tailored interventions for optimal patient care.

## AUTHOR CONTRIBUTIONS

**Sameer Abdul Rauf:** Conceptualization; resources; writing – original draft; writing – review and editing. **Hussain Haider Shah:** Conceptualization; supervision; writing – original draft; writing – review and editing. **Rahul Khatri:** Writing – original draft; writing – review and editing. **Mansoor Ul Haq:** Writing – original draft; writing – review and editing. **Tirth Dave:** Project administration; supervision; validation; writing – original draft; writing – review and editing. **Javaria Parwez Ali:** Writing – original draft; writing – review and editing. **Syed Khizar Ali:** Writing – original draft; writing – review and editing.

## FUNDING INFORMATION

None.

## CONFLICT OF INTEREST STATEMENT

None declared.

## ETHICAL APPROVAL

The ethical approval was not required for the case report as per the country's guidelines.

## CONSENT

Written informed consent was obtained from the patient to publish this report in accordance with the journal's patient consent policy.

## Data Availability

The data that support the findings of this article are available from the corresponding author upon reasonable request.
